# Cardiac features and effects of enzyme replacement therapy in Taiwanese patients with Mucopolysaccharidosis IVA

**DOI:** 10.1186/s13023-018-0883-6

**Published:** 2018-08-29

**Authors:** Hsiang-Yu Lin, Ming-Ren Chen, Shan-Miao Lin, Chung-Lieh Hung, Dau-Ming Niu, Chih-Kuang Chuang, Shuan-Pei Lin

**Affiliations:** 1 Department of Medicine, Mackay Medical College, New Taipei City, Taiwan; 2 Department of Pediatrics, Mackay Memorial Hospital, No.92, Sec. 2, Chung-Shan North Road, Taipei, 10449 Taiwan; 3 Department of Medical Research, Mackay Memorial Hospital, 92 Chung-Shan N. Rd., Sec. 2, Taipei, 10449 Taiwan; 4 Mackay Junior College of Medicine, Nursing and Management, Taipei, Taiwan; 5 Department of Medical Research, China Medical University Hospital, China Medical University, Taichung, Taiwan; 6 Division of Cardiology, Department of Internal Medicine, Mackay Memorial Hospital, Taipei, Taiwan; 7 Department of Pediatrics, Taipei Veterans General Hospital, Taipei, Taiwan; 8 College of Medicine, Fu-Jen Catholic University, Taipei, Taiwan; 9 Department of Infant and Child Care, National Taipei University of Nursing and Health Sciences, Taipei, Taiwan

**Keywords:** Cardiac, Echocardiography, Electrocardiography, Enzyme replacement therapy, Mucopolysaccharidosis IVA

## Abstract

**Background:**

Cardiac abnormalities have been observed in patients with mucopolysaccharidosis (MPS) of any type, with the most documented abnormalities being valvular heart disease and cardiac hypertrophy. However, few studies have focused on the cardiac features of MPS IVA.

**Methods:**

We reviewed the medical records, echocardiograms, and electrocardiograms of 32 Taiwanese patients with MPS IVA (16 males and 16 females; median age, 10.8 years; age range, 1.1 to 29.1 years) as well as the echocardiographic data of six patients who received enzyme replacement therapy (ERT) for 3–6 years.

**Results:**

Echocardiographic examinations (*n* = 32) revealed mean *z* scores of left ventricular mass index (LVMI), interventricular septum diameter in diastole (IVSd), left ventricular posterior wall diameter in diastole (LVPWd), and aortic diameter of 0.94, 2.70, 0.39, and 3.26, respectively. *Z* scores > 2 were identified in 25%, 50%, 29%, and 69% of the LVMI, IVSd, LVPWd, and aortic diameter values, respectively. Diastolic dysfunction [reversed ratio between early and late (atrial) ventricular filling velocity (E/A ratio < 1)] was identified in four patients (13%), however, the ejection fraction was normal (50–75%) in all of the patients. Sixteen patients (50%) had valvular heart disease and most were of mild degree. Fourteen (44%) had valvular stenosis, and 10 (31%) had regurgitation. The *z* scores of LVMI, IVSd, LVPWd, and aortic diameter, the severity scores of aortic stenosis and regurgitation, and the existence of a thickened interventricular septum were all positively correlated with increasing age (*p* < 0.05). For the 14 patients with valve thickening, the *z* scores of LVMI, IVSd and aortic diameter were all larger than those of the 18 patients without valve thickening (*p* < 0.05). For two patients who started ERT at a younger age (1.4 and 2.8 years, respectively), the *z* scores for LVMI, IVSd, and LVPWd all decreased after ERT.

**Conclusions:**

A large proportion of the patients with MPS IVA had valvular heart disease and cardiac hypertrophy. Cardiac abnormalities worsened with increasing age in accordance with the progressive nature of this disease. ERT appeared to be effective in stabilizing or reducing cardiac hypertrophy, and better results may have been associated with starting ERT at a younger age.

**Electronic supplementary material:**

The online version of this article (10.1186/s13023-018-0883-6) contains supplementary material, which is available to authorized users.

## Background

Mucopolysaccharidoses (MPSs; OMIM 252700) are composed of a group of rare genetic disorders caused by deficiencies in specific lysosomal enzymes and involve the sequential degradation of glycosaminoglycans (GAGs) which cause substrate accumulation in various cells and tissues, and progressive multi-organ dysfunction. Seven distinct types of MPS disorders (I, II, III, IV, VI, VII, and IX) with 11 specific lysosomal enzyme deficiencies have been described [1, 2]. The onset and severity of cardiovascular defects differ in each type of MPS, with the most documented abnormalities being cardiac valve thickening, valvular regurgitation and stenosis, and cardiac hypertrophy [3–10]. It has been reported that valve defects and cardiomyopathy result from GAG accumulation in the spongiosa of cardiac valves, myointima of coronary arteries, and myocardium [11]. Mitral or aortic leaflet thickening and calcification may lead to stenosis or regurgitation, and deformities in cardiac structures resulting in cardiac dysfunction can significantly increase morbidity and mortality in patients with MPS [12–14].

Among the different types of MPS diseases, MPS IVA (Morquio A syndrome; OMIM 253000) is a relatively rare autosomal recessive inherited disorder caused by *N*-acetylgalactosamine-6-sulfatase (GALNS) deficiency. MPS IVA can lead to excessive lysosomal storage of GAGs, keratan sulfate and chondroitin-6-sulfate in various tissues and organs. Patients with MPS IVA generally appear unaffected at birth, but may develop multiple clinical manifestations including systemic skeletal chondrodysplasia, short stature, valvular heart disease, corneal clouding, hearing loss, malformation of the thorax that impairs respiratory function, joint abnormalities, odontoid hypoplasia and ligamentous laxity, cervical spinal instability and potentially spinal cord compression [1, 2, 15]. Several studies have described the prevalence of cardiac defects in the more common types of MPS disease [3–10], however, few studies have focused on the cardiac features of MPS IVA [16–21]. Elosulfase alfa (Vimizim®; BioMarin Pharmaceutical Inc., Novato, CA, USA) is a recombinant human GALNS associated with increased endurance, reduced urine keratan sulfate and acceptable safety profile, and it has been approved as enzyme replacement therapy (ERT) for MPS IVA [22, 23]. Nonetheless, information regarding the cardiac characteristics and the long-term cardiac outcomes of ERT for patients with MPS IVA is limited [10]. The purpose of this study was to investigate the cardiologic features of Taiwanese patients with MPS IVA, and evaluate the impact of ERT on cardiac structure and function.

## Methods

### Study population

We retrospectively reviewed the medical records, echocardiograms, and electrocardiograms of 32 Taiwanese patients with MPS IVA (16 males and 16 females; median age, 10.8 years; age range, 1.1 to 29.1 years) at Mackay Memorial Hospital between July 1999 and April 2018. The diagnosis of MPS IVA was confirmed by two-dimensional electrophoresis of urinary GAGs and GALNS activity assays of serum, leukocytes and/or skin fibroblasts [24]. The echocardiographic data of six patients with MPS IVA who received 2.0 mg/kg/week intravenous elosulfase alfa at Mackay Memorial Hospital for 3–6 years were reviewed. The relationships between age and each echocardiographic parameter were also analyzed. None of the patients had received ERT or a hematopoietic stem cell transplantation at baseline. Written informed consent for cardiac evaluations and ERT was obtained from a parent for children and from the patients if they were over 18 years of age. The study was approved by the Ethics Committee of Mackay Memorial Hospital, Taipei, Taiwan.

### Measurements of cardiovascular parameters

A Philips Sonos 5500/7500 ultrasound system (Andover, MA, USA) equipped with electronic transducers from 2 to 8 MHz was used. Data were digitally stored and analyzed by one experienced cardiologist (MRC) to minimize inter-observer variations. Diastolic and systolic diameters were measured using M-mode, and the systolic function of the left ventricle was evaluated according to the ejection fraction obtained using the Teichholz method [V = 7D^3^/(2.4 + D), where V = left ventricular (LV) volume and D = LV diameter] [25]. An ejection fraction ≧ 55% and shortening fraction ≧ 28% were considered to be normal. Diastolic filling was established using the E/A ratio by measuring mitral-inflow as determined by pattern-peak early filling (E) and late filling (A) velocities, and systolic function using the shortening fraction [26]. A reversed E/A ratio (E/A ratio < 1) was considered to indicate diastolic dysfunction. The severity of valvular stenosis and regurgitation were estimated and graded as follows: 0 (none), 1 (mild), 2 (moderate), and 3 (severe) based on the European Society of Cardiology guidelines [9, 10, 27, 28]: mild AS = valve area > 1.5 cm^2^ and mean gradient < 30 mmHg; moderate AS = valve area between 1.0–1.5 cm^2^ and mean gradient between 30 and 50 mmHg; severe AS = valve area < 1.0 cm^2^ and mean gradient > 50 mmHg; mild MS = valve area > 1.5 cm^2^ and mean gradient < 5 mmHg; moderate MS = valve area between 1.0–1.5 cm^2^ and mean gradient between 5 and 10 mmHg; severe MS = valve area < 1.0 cm^2^ and mean gradient > 10 mmHg.

Data on left ventricular mass index (LVMI), right ventricular end diastolic dimension (RVDd), thicknesses of the interventricular septum diameters in diastole (IVSd) and in systole (IVSs), left ventricular internal diameter in diastole (LVIDd) and in systole (LVIDs), thicknesses of the left ventricular posterior wall diameter in diastole (LVPWd) and in systole (LVPWs), aortic diameter, and left atrial dimension (LAD) obtained by echocardiographic assessments [29] were recorded. The measurement of the aorta was made on sinus by leading edge to leading edge. Thickened valves were defined according to the study of Sahasakul et al. [30]. These values were compared with normal values according to the study of Kampmann et al. [31]. Left ventricular mass (LVM) was calculated according to the American Society of Echocardiography simplified cubed equation. LVM was indexed (LVMI) by height^2.7^ to normalize the size of the heart to body size. The LVMI was also calculated using the Devereux formula and indexed by body surface area with normal values according to the report of Poutanen et al. [32]. All of the aforementioned echocardiographic values were transformed into a *z* score derived by subtracting the mean reference value from an individual observed value, and then dividing the difference by the standard deviation from the reference value. A *z* score > 2 was considered to be abnormal. In addition, 27 patients also had available electrocardiographic (ECG) data.

### Data analysis and statistics

The sex, age, height, weight, body mass index, and body surface area at the time of the study were recorded in each patient. Descriptive statistics, including means and standard deviations of all echocardiographic values were calculated. The relationships between age and different echocardiographic parameters were determined using Pearson’s correlation coefficient (*r*), and significance was tested using Fisher’s *r–z* transformations. We compared biometric characteristics and echocardiographic assessments between patients without valve thickening versus those with valve thickening using the Student’s t-test for continuous variables and Fisher’s exact test for categorical variables. Two-tailed *p*-values were computed. All statistical analyses were performed using SPSS version 11.5 (SPSS Inc., Chicago, Illinois, USA), and differences with *p* < 0.05 were considered to be statistically significant.

## Results

**Fig. 1:**
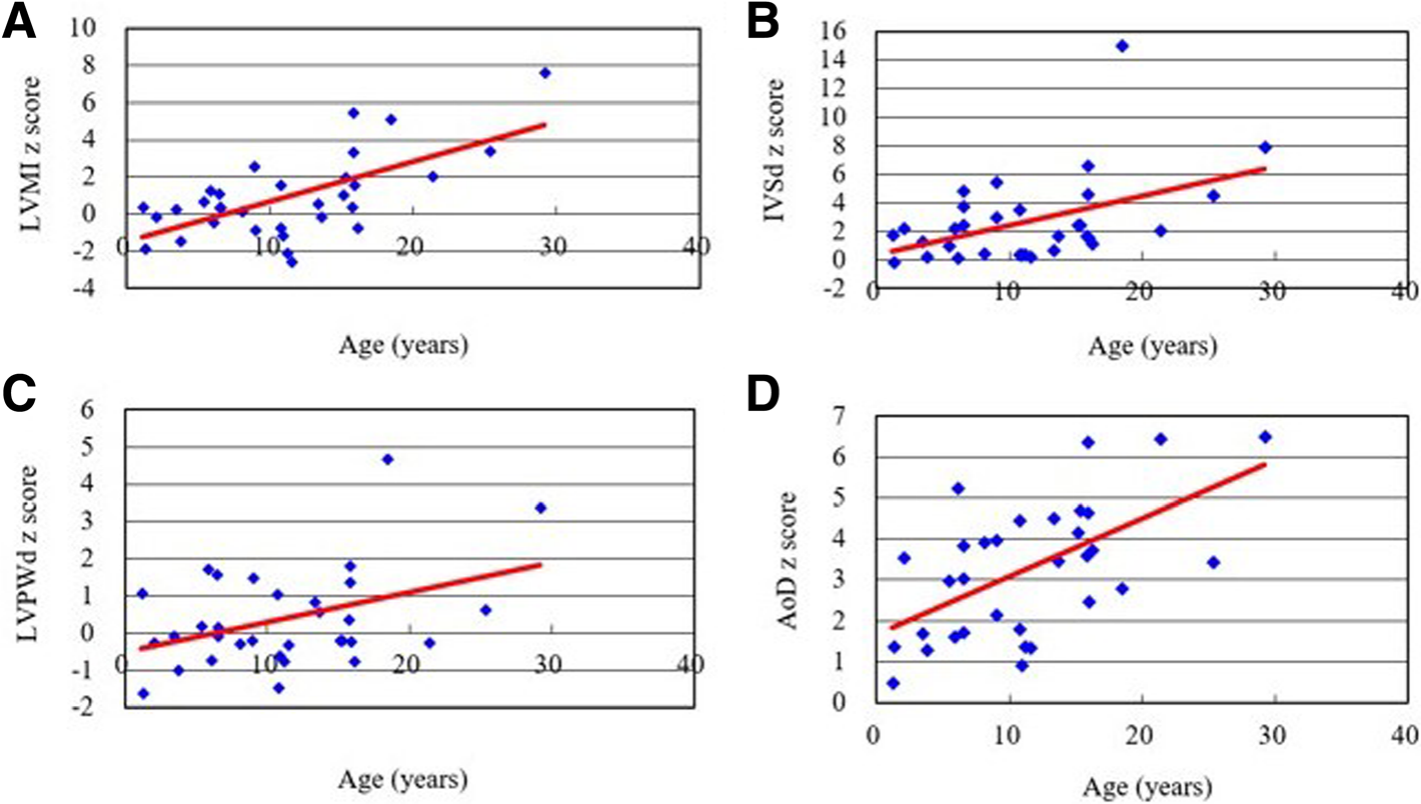
The relationships between age and *z* scores of LVMI (**a**), IVSd (**b**), LVPWd (**c**), and AoD (**d**) of the 32 patients with MPS IVA (all *p* < 0.05). MPS, mucopolysaccharidosis; LVMI, left ventricular mass index; IVSd, interventricular septum thickness in diastole; LVPWd, left ventricular posterior wall thickness in diastole; AoD, aortic diameter

**Fig. 2:**
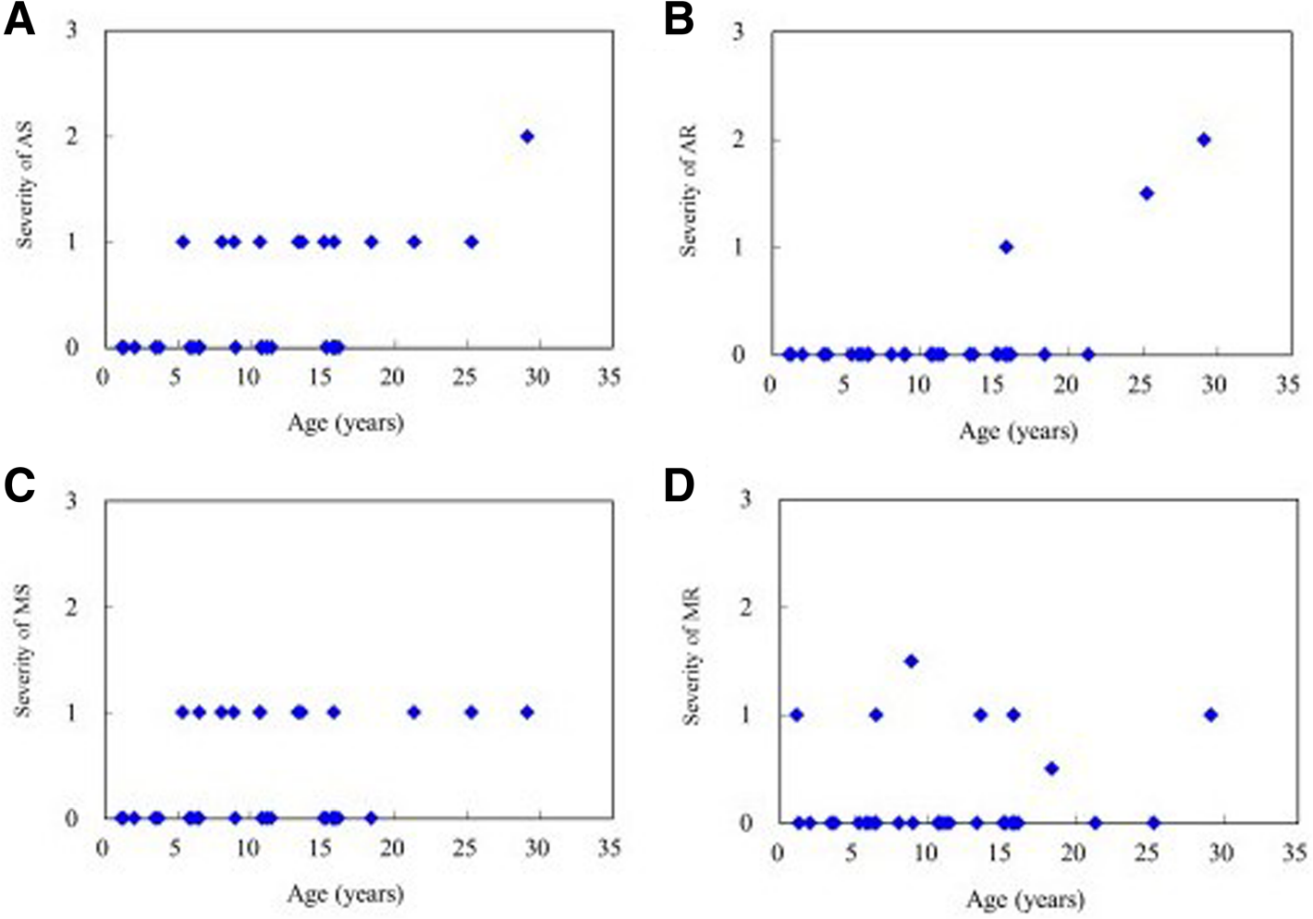
The relationships between age and severity of cardiac valve abnormalities in the 32 patients with MPS IVA (severity score: 3: severe, 2: moderate, 1: mild, 0: normal). (**a**) AS, aortic stenosis; (**b**) AR, aortic regurgitation; (**c**) MS, mitral stenosis; (**d**) MR, mitral regurgitation

Table 1 showed the baseline clinical, echocardiographic and electrocardiographic features of 32 patients with MPS IVA. Echocardiographic examinations (*n* = 32) revealed that the mean *z* scores of LVMI, IVSd, LVPWd, and aortic diameter were 0.94, 2.70, 0.39, and 3.26, respectively (Table 2). *Z* scores > 2 were identified in 25%, 50%, 29%, and 69% of the LVMI, IVSd, LVPWd, and aortic diameter values, respectively. Diastolic dysfunction (E/A ratio < 1) was identified in four patients (13%), however, the ejection fraction (reference ≧ 55%) and shortening fraction (reference ≧ 28%) values were all normal and revealed normal systolic function. Sixteen patients (50%) had valvular heart disease and most were of mild degree. Fourteen (44%) had valvular stenosis, and 10 (31%) had regurgitation. Valvular stenosis was mild in all but one case who was adult (No. 32) and that regurgitation was also mild in all but three cases. No one had stenosis under the age of 5 years. Fourteen (44%) and 12 (38%) patients had mitral valve disease or aortic valve disease, respectively. Two (6%) patients had tricuspid valve disease, however none of the patients had pulmonary valve disease. The most prevalent cardiac valve abnormalities were mitral stenosis (38%) and aortic stenosis (38%), followed by mitral regurgitation (22%), aortic regurgitation (9%), and tricuspid regurgitation (6%). Overall, 22% and 31% of the patients had mitral valve prolapse and a thickened interventricular septum, respectively (Table 3). The *z* scores of LVMI, IVSd, IVSs, LVPWd, LVPWs, and aortic diameter, the severity scores of aortic stenosis and regurgitation, and the existence of a thickened interventricular septum were all positively correlated with increasing age (*p* < 0.05) (Tables 2 and 3, Figs. 1 and 2). The mean ages of the patients with and without valve thickening were 14.4 and 8.7 years, respectively. The mean LVMI values of the patients with and without valve thickening were 80.9 and 53.9 g/m^2.7^, respectively. For the 14 patients with valve thickening, the *z* scores of LVMI, IVSd, IVSs, LVPWs, and aortic diameter were all larger than those of the 18 patients without valve thickening (*p* < 0.05) (Table 4). Twenty-one patients (78%, *n* = 27) had abnormal ECG findings, the most common of which was the presence of sinus tachycardia (37%), followed by sinus arrhythmia (33%), right or left axis deviation (19%), and cardiac enlargement (11%). The ECG abnormalities were usually of minor clinical significance. For the seven patients with MPS IVA (age range, 1.4 to 25.8 years) treated with weekly intravenous infusions of elosulfase alfa (2.0 mg/kg) for 3–6 years, echocardiography showed a decrease in mean LVMI *z* score from 0.77 to 0.73, a decrease in mean IVSd *z* score from 2.42 to 2.17, and a decrease in mean LVPWd *z* score from 0.41 to − 0.50. For the two patients who started ERT at a younger age (1.4 years and 2.8 years of age, respectively), the *z* scores for LVMI, IVSd, and LVPWd all decreased after ERT (Table 5). However, ERT seemed to have little or no effect on valvular heart disease (Additional file 1: Table S1).

**Table 1:** Baseline clinical, echocardiographic and electrocardiographic features of 32 patients with MPS IVA

No.	Gender	Age (years)	LVMI (z score)	RVDd (z score)	IVSd (z score)	IVSs (z score)	LVIDd (z score)	LVIDs (z score)	LVPWd (z score)	LVPWs (z score)	AoD (z score)	LAD (z score)	EF	SF (%)	Reversed E/A ratio	MS	MR	AS	AR	MVP	Thick IVS	Electrocardiographic features
1	M	1.1	0.40	-0.07	1.83	-0.32	-0.18	-0.70	1.09	-1.22	0.52	-0.86	0.74	42%	-	0	1	0	0	+	-	Normal
2	M	1.3	-1.83	1.56	-0.16	-1.03	1.08	1.85	-1.61	-0.17	1.36	-0.10	0.60	31%	-	0	0	0	0	-	-	Sinus tachycardia
3	M	2.0	-0.11	2.18	2.25	0.34	-0.32	-1.55	-0.25	0.26	3.56	0.14	0.80	47%	-	0	0	0	0	-	-	NA
4	F	3.4	0.33	3.18	1.28	0.36	0.46	-0.58	-0.07	-1.36	1.71	-1.40	0.76	43%	-	0	0	0	0	-	-	Sinus tachycardia, RAD
5	F	3.7	-1.40	0.31	0.20	-0.23	-0.19	-0.60	-0.97	-1.06	1.31	1.36	0.74	41%	-	0	0	0	0	-	-	Normal
6	M	5.3	0.69	2.71	0.99	0.29	1.22	0.05	0.21	2.00	3.00	1.64	0.76	43%	-	1	0	1	0	-	-	Sinus tachycardia, RAD
7	M	5.8	1.34	0.50	2.24	-0.03	0.14	0.49	1.73	-2.64	1.64	-1.30	0.65	34%	-	0	0	0	0	-	-	Sinus tachycardia, septal hypertrophy
8	M	6.0	-0.43	0.98	0.14	-1.19	1.64	1.15	-0.71	-0.92	5.26	-0.05	0.71	40%	-	0	0	0	0	-	-	LAD
9	F	6.4	1.13	0.59	3.77	-0.52	-0.90	-0.26	1.60	-1.33	1.73	0.26	0.66	35%	-	0	0	0	0	-	-	Sinus arrhythmia
10	F	6.5	0.42	3.73	2.48	1.55	0.37	0.15	0.16	0.64	3.06	-0.24	0.69	38%	-	1	1	0	0	+	-	Normal
11	M	6.5	0.36	4.59	4.89	2.13	-1.00	-0.09	-0.07	-2.24	3.86	0.30	0.62	32%	-	0	0	0	0	-	-	RAD
12	F	8.0	0.17	3.50	0.45	0.03	0.83	0.31	-0.28	-0.82	3.94	1.12	0.71	40%	-	1	0	1	0	-	-	Sinus arrhythmia, low voltage
13	M	8.9	2.61	NA	5.47	1.56	1.43	-0.36	-0.19	1.52	4.00	4.80	0.79	46%	-	1	1.5	1	0	-	+	Sinus arrhythmia
14	M	9.0	-0.82	0.45	3.00	0.79	-1.29	-1.00	1.51	-0.82	2.18	1.88	0.69	37%	+	0	0	0	0	-	+	Sinus tachycardia, LAD
15	F	10.7	-0.72	0.59	3.51	1.05	-2.46	-2.92	1.06	-1.71	1.82	1.44	0.80	47%	-	1	0	1	0	+	+	Sinus tachycardia
16	M	10.7	1.62	-0.84	0.37	2.65	4.20	2.13	-1.45	-0.12	4.47	-0.21	0.76	45%	-	1	0	0	0	-	+	Sinus arrhythmia
17	F	10.8	-1.12	3.11	0.39	-0.46	-0.06	-0.08	-0.59	-0.14	0.94	-1.00	0.68	37%	-	0	0	0	0	-	-	Normal
18	F	11.1	-2.08	1.30	0.37	-1.39	-0.79	0.50	-0.75	-2.01	1.39	0.71	0.55	28%	-	0	0	0	0	-	-	Normal
19	F	11.5	-2.52	2.23	0.21	-0.27	-1.46	-2.40	-0.29	-0.32	1.36	-1.30	0.80	47%	-	0	0	0	0	-	-	Sinus arrhythmia
20	F	13.3	0.61	0.41	0.68	0.57	0.40	-0.19	0.86	0.48	4.53	1.20	0.73	41%	-	1	0	1	0	-	-	Sinus arrhythmia
21	F	13.6	-0.14	2.45	1.73	1.45	-0.50	-0.26	0.59	0.16	3.47	0.26	0.69	38%	-	1	1	1	0	+	-	Sinus arrhythmia
22	F	15.1	1.08	0.60	2.50	-0.46	0.48	-0.44	-0.18	0.76	4.18	0.70	0.74	42%	-	0	0	1	0	+	-	Sinus tachycardia
23	F	15.3	2.01	NA	2.45	1.94	2.32	0.16	-0.18	-0.58	4.71	0.03	0.78	46%	-	0	0	0	0	-	+	Sinus arrhythmia
24	M	15.7	0.45	0.73	1.73	1.45	1.54	0.04	0.37	0.41	3.64	-0.75	0.77	44%	-	0	0	0	0	-	-	NA
25	M	15.8	5.49	-0.13	4.63	6.26	4.03	1.08	1.84	0.76	4.65	-0.10	0.79	47%	-	0	0	0	0	-	-	NA
26	F	15.8	3.36	2.23	6.60	1.84	0.67	-0.21	1.37	1.67	6.40	0.34	0.76	43%	+	1	1	1	1	+	+	Sinus tachycardia, LAE
27	M	15.9	1.58	-0.32	1.60	0.88	3.20	1.75	-0.22	0.13	2.48	1.59	0.68	39%	-	0	0	0	0	-	-	NA
28	F	16.1	-0.72	1.20	1.14	-1.47	-0.27	-0.29	-0.75	-1.45	3.76	-0.68	0.67	37%	-	0	0	0	0	-	-	Sinus tachycardia
29	F	18.4	5.14	-2.50	15.07	5.20	-0.95	-0.65	4.71	1.62	2.82	0.04	0.68	36%	-	0	0.5	1	0	-	+	NA
30	M	21.3	2.08	-2.68	2.13	4.67	2.15	-0.96	-0.24	0.69	6.47	-1.16	0.85	54%	+	1	0	1	0	+	+	Sinus tachycardia, RAE
31	M	25.3	3.43	-0.13	4.51	2.29	1.02	-0.61	0.65	2.37	3.44	-1.90	0.78	46%	-	1	0	1	1.5	-	+	Sinus arrhythmia, IRBBB
32	M	29.1	7.65	3.57	8.00	2.84	1.03	-0.60	3.39	2.41	6.53	0.17	0.78	46%	+	1	1	2	2	-	+	Normal

*MPS* mucopolysaccharidosis, *LVMI* left ventricular mass index, *RVDd* right ventricular end diastolic dimension, *IVSd* interventricular septum thickness in diastole, *IVSs* interventricular septum thickness in systole, *LVIDd* left ventricular internal diameter in diastole, *LVIDs* left ventricular internal diameter in systole, *LVPWd* left ventricular posterior wall thickness in diastole, *LVPWs* left ventricular posterior wall thickness in systole, *AoD* aortic diameter, *LAD* left atrial dimension, *EF* ejection fraction, *SF* shortening fraction, *E/A* ratio between early and late (atrial) ventricular filling velocity, *MS* mitral stenosis, *MR* mitral regurgitation, *AS* aortic stenosis, *AR* aortic regurgitation, *MVP* mitral valve prolapse, *IVS* interventricular septum, *NA* not available, *RAD* right axis deviation, *LAD* left axis deviation, *LAE* left atrial enlargement, *RAE* right atrial enlargement, *IRBBB* irregular bundle branch block. Severity of valvular stenosis and regurgitation (MS, MR, AS, AR) were estimated and graded on the following scores: 0 (none), 1 (mild), 2 (moderate), and 3 (severe)

**Table 2:** The values of echocardiographic parameters and their relationships with age in the 32 patients with MPS IVA

Echocardiographic parameters	LVMI (z score)	RVDd (z score)	IVSd (z score)	IVSs (z score)	LVIDd (z score)	LVIDs (z score)	LVPWd (z score)	LVPWs (z score)	AoD (z score)	LAD (z score)
Mean (SD)	0.94	1.20	2.70	1.02	0.56	−0.16	0.39	−0.10	3.26	0.22
	(2.24)	(1.74)	(3.02)	(1.85)	(1.52)	(1.06)	(1.32)	(1.34)	(1.64)	(1.29)
*r* value (echocardiographic parameter versus age)	0.647	−0.276	0.458	0.544	0.248	−0.085	0.409	0.563	0.582	−0.156
*p* value	*p* < 0.01	*p* > 0.05	*p* < 0.01	*p* < 0.05	*p* > 0.05	*p* > 0.05	*p* < 0.05	*p* < 0.01	*p* < 0.01	*p* > 0.05

*MPS*, mucopolysaccharidosis; *LVMI*, left ventricular mass index; *RVDd*, right ventricular end diastolic dimension; *IVSd*, interventricular septum thickness in diastole; *IVSs,* interventricular septum thickness in systole; *LVIDd,* left ventricular internal diameter in diastole; *LVIDs*, left ventricular internal diameter in systole; *LVPWd*, left ventricular posterior wall thickness in diastole; *LVPWs*, left ventricular posterior wall thickness in systole; *AoD*, aortic diameter; *LAD*: left atrial dimension; *SD,* standard deviation

**Table 3:** Echocardiographic features of the 32 patients with MPS IVA and the relationships between severity of cardiac valve abnormalities and age

Echocardiographic features	MS	MR	AS	AR	TS	TR	PS	PR	MVP	Thick IVS
%	38%	22%	38%	9%	0%	6%	0%	0%	22%	31%
*r* value (severity of cardiac valve abnormalities versus age)	0.329	0.109	0.582	0.638	ND	0.008	ND	ND	0.062	0.530
*p* value	*p*>0.05	*p*>0.05	*p*<0.01	*p*<0.01	*p*>0.05	*p*>0.05	*p*>0.05	*p*>0.05	*p*>0.05	*p*<0.01

*MPS* mucopolysaccharidosis, *MS* mitral stenosis, *MR* mitral regurgitation, *AS* aortic stenosis, *AR* aortic regurgitation, *TS* tricuspid stenosis, *TR* tricuspid regurgitation, *PS* pulmonary stenosis, *PR* pulmonary regurgitation, *MVP* mitral valve prolapse, *IVS* interventricular septum

**Table 4:** Biometric characteristics and echocardiographic assessments of the 32 patients with MPS IVA with and without valvular thickening

Echocardiographic parameter	Without valvular thickening (n=18)	With valvular thickening (n=14)	*p* value
Gender (male/female)	10/8	6/8	0.492
Age (years)	8.7 ± 5.4	14.4 ± 7.1	**0.015**
Height (*z* score)	-5.00 ± 3.20	-8.92 ± 3.21	**0.002**
Weight (*z* score)	-1.83 ± 1.13	-2.63 ± 0.93	**0.042**
BMI (*z* score)	0.02 ± 1.06	0.53 ± 1.24	0.219
BSA (m^2^)	0.70 ± 0.25	0.70 ± 0.08	0.956
LVMI (g/m^2.7^)	53.9 ± 24.1	80.9 ± 46.6	**0.042**
LVMI (*z* score)	0.11 ± 1.86	2.00 ± 2.30	**0.015**
SF (%) (normal ≧28)	39.3 ± 6.0	43.2 ± 4.6	0.051
RVDd (*z* score)	1.32 ± 1.35 (n=17)	1.05 ± 2.20 (n=13)	0.683
IVSd (*z* score)	1.78 ± 1.54	3.89 ± 3.99	**0.047**
IVSs (*z* score)	0.40 ± 1.81	1.82 ± 1.63	**0.029**
LVIDd (*z* score)	0.44 ± 1.56	0.71 ± 1.51	0.633
LVIDs (*z* score)	-0.03 ± 1.08	-0.33 ± 1.06	0.441
LVPWd (*z* score)	0.09 ± 1.03	0.76 ± 1.58	0.159
LVPWs (*z* score)	-0.82 ± 0.95	0.83 ± 1.20	**0.0001**
AoD (*z* score)	2.56 ± 1.47	4.15 ± 1.44	**0.005**
LAD (*z* score)	-0.07 ± 0.99	0.58 ± 1.56	0.158
EF	0.70 ± 0.07	0.75 ± 0.05	0.052
With diastolic dysfunction (MV E/A<1)	n=1 (6%)	n=3 (21%)	0.189
MV E/A (normal≧1)	1.41 ± 0.27 (n=12)	1.16 ± 0.26 (n=14)	**0.025**
TV E/A (normal≧1)	1.30 ± 0.36 (n=11)	1.31 ± 0.32 (n=10)	0.928

*MPS* mucopolysaccharidosis, *BMI* body mass index, *BSA* body surface area, *LVMI* left ventricular mass index, *SF* shortening fraction, *RVDd* right ventricular end diastolic dimension, *IVSd* interventricular septum thickness in diastole, *IVSs* interventricular septum thickness in systole, *LVIDd* left ventricular internal diameter in diastole, *LVIDs* left ventricular internal diameter in systole, *LVPWd* left ventricular posterior wall thickness in diastole, *LVPWs* left ventricular posterior wall thickness in systole, *AoD* aortic diameter, *LAD* left atrial dimension, *EF* ejection fraction, *MV* mitral valve, *E/A* ratio between early and late (atrial) ventricular filling velocity, *TV* tricuspid valve

**Table 5:** Baseline and follow-up echocardiographic parameters of seven Taiwanese patients with MPS IVA who received ERT for 3-6 years

No.	Age at start of ERT (years)	ERT duration (years)	LVMI (*z* score)		IVSd (*z* score)		LVPWd (*z* score)	
			Baseline	Follow-up	Baseline	Follow-up	Baseline	Follow-up
1	1.4	6.0	0.44	-1.22	1.8	0.5	1.1	-0.5
2	2.8	3.0	0.46	-1.24	2.0	1.0	0.2	-2.2
6	7.3	4.0	2.47	2.64	1.2	2.2	-0.1	-0.2
11	13.9	3.0	-1.46	0.05	5.2	3.1	0.9	-0.5
20	14.9	4.1	1.09	1.47	4.3	1.9	0.4	0.0
23	16.8	4.1	-1.41	0.25	-0.1	0.5	-1.9	0.1
31	25.8	4.4	3.8	3.17	2.4	6.0	2.3	-0.2
Mean	11.8	4.1	0.77	0.73	2.42	2.17	0.41	-0.50
SD	8.6	1.0	1.92	1.76	1.81	1.94	1.29	0.78

*MPS* mucopolysaccharidosis, *ERT* enzyme replacement therapy, *LVMI* left ventricular mass index, *IVSd* interventricular septum thickness in diastole, *LVPWd* left ventricular posterior wall thickness in diastole, *SD* standard deviation

## Discussion

To the best of our knowledge, this is the first report to describe the cardiac characteristics of Asian patients with MPS IVA and compare them with normal values derived from a population that included young adults according to the study of Kampmann et al. [31], and to measure the effects of ERT on cardiac structure and function in these patients. Few studies have focused on the cardiac features of patients with MPS IVA [16–21]. Compared with the other types of MPS diseases, due to the severe skeletal deformities and dysmorphic stature of patients with MPS IVA, non-skeletal manifestations, including cardiovascular and respiratory impairment, have received relatively little attention [33]. Our results demonstrated that most of the patients with MPS IVA had cardiac hypertrophy, aortic dilatation, increased thickness of the interventricular septum, normal systolic function, and mildly valvular heart disease. The cardiac abnormalities in these patients worsened with increasing age in accordance with the progressive nature of this disease. Our results are consistent with those of previous studies from Caucasian populations [16, 17]. In addition, ERT appeared to be effective in stabilizing cardiac hypertrophy, and better results may have been associated with starting ERT at a younger age.

Most of the patients with MPS IVA in this study (97%) had cardiac abnormalities, and only one 1.3-year-old male patient had normal cardiac features in both echocardiography and ECG. Echocardiographic assessments revealed mean *z* scores of LVMI, IVSd, LVPWd, and aortic diameter of 0.94, 2.70, 0.39, and 3.26, respectively. *Z* scores > 2 were identified in 25%, 50%, 29%, and 69% of the LVMI, IVSd, LVPWd, and aortic diameter values, respectively. Kampmann et al. [16] reported thickened left ventricles and aortic root extensions in patients with MPS IVA, and Bolourchi et al. [34] reported that patients with MPS IVA had the highest prevalence of aortic root dilatation (62.5%) among all MPS types. Our results were consistent with theirs. Although left ventricular systolic function was normal in all of our patients, diastolic dysfunction with a reversed E/A ratio (< 1) was identified in four patients (13%). The general consensus in previous studies has been that abnormal catabolism of dermatan sulfate in patients with MPS I, II and VI leads to the accumulation of dermatan-sulfated GAGs in cardiac valves, resulting in valvular thickening and other cardiac defects [6, 7]. The main storage products of MPS IVA are keratan sulfate and chondroitin sulfate. Therefore, cardiac lesions may be less prominent in MPS IVA than in MPS I, II, and VI. In our cohort, substantial cardiac hypertrophy, aortic dilatation, as well as valvular stenosis and regurgitation were still present in the patients with MPS IVA, and the severity also worsened with increasing age.

Deformed mitral or aortic valves were more commonly found than tricuspid or pulmonary valves in our cohort. Previous studies have reported that left-sided valves are much more commonly involved than right-sided valves in patients with MPS IVA [17, 18], which is consistent with our results. There were varying degrees of valvular deformities, although most of the patients had mild stenosis or regurgitation [3, 7]. However, mitral or aortic stenosis was more common than regurgitation in our cohort, which is different to the results of most reports in Caucasian populations [7]. Further studies are warranted to clarify whether this difference is caused by actual ethnic differences or an artifact of a limited sample size.

For the 14 patients with valve thickening, the *z* scores of LVMI, IVSd, IVSs, LVPWs, and aortic diameter were all larger than those of the 18 patients without valve thickening (*p* < 0.05). This suggests that the patients with valve thickening required a higher cardiac muscle mass and higher heart rate-normalized work index to keep cardiac output constant, which is consistent with a previous report [16].

Seventy-eight percent of our ECGs showed abnormal findings, although the clinical significance was minor. The most common finding was sinus tachycardia (37%), which has frequently been observed in patients with MPS IVA. Although the underlying mechanism remains unclear, it seems to be a physiological reflex to maintain cardiac output in a small-sized heart with impaired filling patterns [16]. Cardiac enlargement was found in 11% of our patients by ECG, which was not consistent with the cardiac hypertrophy identified by echocardiography, possibly owing to the low electric conductance of GAGs [8]. Although ECG has been reported to be an unreliable tool for detecting cardiologic defects in MPS [8], one patient was found to have an irregular bundle branch block by ECG in this cohort. Therefore, we suggest that ECG should remain part of the follow-up examinations of patients with MPS IVA, especially to identify changes in conduction or rhythm abnormalities.

Cardiac disease can occur insidiously and lead to early mortality in patients with MPS IVA [13]. Comprehensive physical examinations, 12-lead complete ECG, and echocardiography when MPS is diagnosed, followed by routine cardiac function follow-up are important [35, 36]. Without regular cardiac monitoring, cardiac lesions may remain undetected because of a lack of physical activity due to skeletal dysplasia and pulmonary function impairment. If ventricular arrhythmia is detected, 24-h Holter monitoring should also be considered.

In our cohort, the mean *z* scores of LVMI, IVSd and LVPWd all decreased after ERT. This suggests that ERT has some effect on GAG accumulation in the cardiac tissue of patients with MPS, and thus is effective in reducing cardiac hypertrophy. However, ERT seemed to have little or no effect on valvular heart disease, which is consistent with previous studies [8, 10, 11, 37–40]. Braunlin et al. [39] and Kampmann et al. [40] reported that ERT may have better long-term results when started at an early age for patients with MPS VI, which is consistent with our results. Several sibling control studies have also reported that ERT may prevent or delay the development of valvular heart disease when started early in life [41–45]. As ERT appears to arrest rather than improve valvular heart disease, starting treatment early may be indicated. The increasing clinical awareness of MPS disease and increased ability to make a confirmative diagnosis has made an earlier diagnosis possible. Due to the progressive nature of MPS, initiating ERT before the occurrence of irreversible cardiac damage may contribute to a better clinical outcome. Thus, making an early diagnosis through screening programs for newborns or high-risk populations is of major importance [46–48].

### Limitations

As a retrospective and uncontrolled study, there were no untreated control subjects to compare the cardiac effects of ERT with our patients. In addition, the small sample size of patients with MPS IVA and those who received ERT in this cohort reflect the rare nature of this genetic disorder. In addition, both the degree of disease severity and age range (1.1–29.1 years) varied considerably. As a result, studies with larger cohorts and longer follow-up periods are warranted.

## Conclusion

Our results showed that a substantial proportion of the patients with MPS IVA had cardiac hypertrophy, aortic dilatation, and mildly valvular heart disease. The cardiac abnormalities in these patients worsened with increasing age in accordance with the progressive nature of this disease. ERT appeared to be effective in stabilizing or reducing cardiac hypertrophy, and better results may have been associated with starting ERT at a younger age. However, previous sibling studies have suggested that ERT appears to arrest rather than improve valvular heart disease. Therefore, it is very important to make an early diagnosis through screening programs for newborns or high-risk populations in order to initiate ERT before the occurrence of irreversible cardiac damage.

## Additional file

Additional file 1: **Table S1.** Baseline and follow-up echocardiographic assessments for valvular heart disease of seven Taiwanese patients with MPS IVA who received ERT for 3–6 years (severity score: 3: severe, 2: moderate, 1: mild, 0: normal). (DOCX 24 kb)

## Abbreviations

E/A: Ratio between early and late (atrial) ventricular filling velocity
ECG: Electrocardiography
ERT: Enzyme replacement therapy
GAGs: Glycosaminoglycans
GALNS: *N*-acetylgalactosamine-6-sulfatase
IVSd: Interventricular septum thickness in diastole
IVSs: Interventricular septum thickness in systole
LAD: Left atrial dimension
LVIDd: Left ventricular internal diameter in diastole
LVIDs: Left ventricular internal diameter in systole
LVM: Left ventricular mass
LVMI: Left ventricular mass index
LVPWd: Left ventricular posterior wall thickness in diastole
LVPWs: Left ventricular posterior wall thickness in systole
MPS: Mucopolysaccharidosis
RVDd: Right ventricular end diastolic dimension

## References

[1] Neufeld EF, Muenzer J: The mucoplysaccharidoses. In: Scriver CR, Beaudet AL, Sly WS, Valle D, eds; Childs B, Kinzler KW, Vogelstein B, assoc, eds. The Metabolic and Molecular Bases of Inherited Disease, 8th edn. New York: McGraw-Hill; 2001, 3421–3452.

[2] Muenzer J (2011). Overview of the mucopolysaccharidoses. Rheumatology (Oxford).

[3] Wippermann CF, Beck M, Schranz D, Huth R, Michel-Behnke I, Jüngst BK (1995). Mitral and aortic regurgitation in 84 patients with mucopolysaccharidoses. Eur J Pediatr.

[4] Mohan UR, Hay AA, Cleary MA, Wraith JE, Patel RG (2002). Cardiovascular changes in children with mucopolysaccharide disorders. Acta Paediatr.

[5] Chen MR, Lin SP, Hwang HK, Yu CH (2005). Cardiovascular changes in mucopolysaccharidoses in Taiwan. Acta Cardiol.

[6] Leal GN, de Paula AC, Leone C, Kim CA (2010). Echocardiographic study of paediatric patients with mucopolysaccharidosis. Cardiol Young.

[7] Braunlin EA, Harmatz PR, Scarpa M, Furlanetto B, Kampmann C, Loehr JP (2011). Cardiac disease in patients with mucopolysaccharidosis: presentation, diagnosis and management. J Inherit Metab Dis.

[8] Brands MM, Frohn-Mulder IM, Hagemans ML, Hop WC, Oussoren E, Helbing WA (2013). Mucopolysaccharidosis: cardiologic features and effects of enzyme-replacement therapy in 24 children with MPS I, II and VI. J Inherit Metab Dis.

[9] Lin SM, Lin HY, Chuang CK, Lin SP, Chen MR (2014). Cardiovascular abnormalities in Taiwanese patients with mucopolysaccharidosis. Mol Genet Metab.

[10] Lin HY, Chuang CK, Chen MR, Lin SM, Hung CL, Chang CY (2016). Cardiac structure and function and effects of enzyme replacement therapy in patients with mucopolysaccharidoses I, II, IVA and VI. Mol Genet Metab.

[11] Braunlin EA, Berry JM, Whitley CB (2006). Cardiac findings after enzyme replacement therapy for mucopolysaccharidosis type I. Am J Cardiol.

[12] Jones SA, Almássy Z, Beck M, Burt K, Clarke JT, Giugliani R (2009). Mortality and cause of death in mucopolysaccharidosis type II-a historical review based on data from the hunter outcome survey (HOS). J Inherit Metab Dis.

[13] Lavery C, Hendriksz C (2015). Mortality in patients with Morquio syndrome a. JIMD Rep.

[14] Lin HY, Chuang CK, Huang YH, Tu RY, Lin FJ, Lin SJ (2016). Causes of death and clinical characteristics of 34 patients with Mucopolysaccharidosis II in Taiwan from 1995-2012. Orphanet J Rare Dis.

[15] Lin HY, Chuang CK, Chen MR, Chiu PC, Ke YY, Niu DM (2014). Natural history and clinical assessment of Taiwanese patients with mucopolysaccharidosis IVA. Orphanet J Rare Dis.

[16] Kampmann C, Abu-Tair T, Gökce S, Lampe C, Reinke J, Mengel E (2016). Heart and cardiovascular involvement in patients with Mucopolysaccharidosis type IVA (Morquio-a syndrome). PLoS One.

[17] John RM, Hunter D, Swanton RH (1990). Echocardiographic abnormalities in type IV mucopolysaccharidosis. Arch Dis Child.

[18] Ireland MA, Rowlands DB (1981). Mucopolysaccharidosis type IV as a cause of mitral stenosis in an adult. Br Heart J.

[19] Nicolini F, Corradi D, Bosio S, Gherli T (2008). Aortic valve replacement in a patient with morquio syndrome. Heart Surg Forum.

[20] Pagel PS, Almassi GH (2009). Perioperative implications of Morquio syndrome in a 31-year-old woman undergoing aortic valve replacement. J Cardiothorac Vasc Anesth.

[21] Barry MO, Beardslee MA, Braverman AC (2006). Morquio's syndrome: severe aortic regurgitation and late pulmonary autograft failure. J Heart Valve Dis.

[22] Hendriksz CJ, Burton B, Fleming TR, Harmatz P, Hughes D, Jones SA (2014). Efficacy and safety of enzyme replacement therapy with BMN 110 (elosulfase alfa) for Morquio a syndrome (mucopolysaccharidosis IVA): a phase 3 randomised placebo-controlled study. J Inherit Metab Dis.

[23] Hendriksz CJ, Parini R, AlSayed MD, Raiman J, Giugliani R, Solano Villarreal ML (2016). Long-term endurance and safety of elosulfase alfa enzyme replacement therapy in patients with Morquio a syndrome. Mol Genet Metab.

[24] Chuang CK, Lin SP, Chung SF (2001). Diagnostic screening for mucopolysaccharidoses by the dimethylmethylene blue method and two dimensional electrophoresis. Zhonghua Yi Xue Za Zhi (Taipei).

[25] Teichholz LE, Kreulen T, Herman MV, Gorlin R (1976). Problems in echocardiographic volume determinations: echocardiographic-angiographic correlations in the presence of absence of asynergy. Am J Cardiol.

[26] Eidem BW, McMahon CJ, Cohen RR, Wu J, Finkelshteyn I, Kovalchin JP (2004). Impact of cardiac growth on Doppler tissue imaging velocities: a study in healthy children. J Am Soc Echocardiogr.

[27] Baumgartner H, Hung J, Bermejo J, Chambers JB, Evangelista A, Griffin BP (2009). Echocardiographic assessment of valve stenosis: EAE/ASE recommendations for clinical practice. J Am Soc Echocardiogr.

[28] Lancellotti P, Tribouilloy C, Hagendorff A, Popescu BA, Edvardsen T, Pierard LA (2013). Recommendations for the echocardiographic assessment of native valvular regurgitation: an executive summary from the European Association of Cardiovascular Imaging. Eur Heart J Cardiovasc Imaging.

[29] Lang RM, Bierig M, Devereux RB, Flachskampf FA, Foster E, Pellikka PA (2005). Recommendations for chamber quantification: a report from the American Society of Echocardiography's guidelines and standards committee and the chamber quantification writing group, developed in conjunction with the European Association of Echocardiography, a branch of the European Society of Cardiology. J Am Soc Echocardiogr.

[30] Sahasakul Y, Edwards WD, Naessens JM, Tajik AJ (1988). Age-related changes in aortic and mitral valve thickness: implications for two-dimensional echocardiography based on an autopsy study of 200 normal human hearts. Am J Cardiol.

[31] Kampmann C, Wiethoff CM, Wenzel A, Stolz G, Betancor M, Wippermann CF (2000). Normal values of M mode echocardiographic measurements of more than 2000 healthy infants and children in Central Europe. Heart.

[32] Poutanen T, Jokinen E (2007). Left ventricular mass in 169 healthy children and young adults assessed by three-dimensional echocardiography. Pediatr Cardiol.

[33] Hendriksz CJ, Harmatz P, Beck M, Jones S, Wood T, Lachman R (2013). Review of clinical presentation and diagnosis of mucopolysaccharidosis IVA. Mol Genet Metab.

[34] Bolourchi M, Renella P, Wang RY. Aortic Root Dilatation in Mucopolysaccharidosis I-VII. Int J Mol Sci. 2016;17(12):E2004. 10.3390/ijms17122004 PMC5187804 27916847

[35] Giugliani R, Harmatz P, Wraith JE (2007). Management guidelines for mucopolysaccharidosis VI. Pediatrics.

[36] Muenzer J, Wraith JE, Clarke LA (2009). International consensus panel on management and treatment of Mucopolysaccharidosis I: Mucopolysaccharidosis I: management and treatment guidelines. Pediatrics.

[37] Wraith JE, Beck M, Lane R, van der Ploeg A, Shapiro E, Xue Y (2007). Enzyme replacement therapy in patients who have mucopolysaccharidosis I and are younger than 5 years: results of a multinational study of recombinant human alpha-L-iduronidase (laronidase). Pediatrics.

[38] Fesslová V, Corti P, Sersale G, Rovelli A, Russo P, Mannarino S (2009). The natural course and the impact of therapies of cardiac involvement in the mucopolysaccharidoses. Cardiol Young.

[39] Braunlin E, Rosenfeld H, Kampmann C, Johnson J, Beck M, Giugliani R (2013). Enzyme replacement therapy for mucopolysaccharidosis VI: long-term cardiac effects of galsulfase (Naglazyme®) therapy. J Inherit Metab Dis.

[40] Kampmann C, Lampe C, Whybra-Trümpler C, Wiethoff CM, Mengel E, Arash L (2014). Mucopolysaccharidosis VI: cardiac involvement and the impact of enzyme replacement therapy. J Inherit Metab Dis.

[41] Gabrielli O, Clarke LA, Bruni S, Coppa GV (2010). Enzyme-replacement therapy in a 5-month-old boy with attenuated presymptomatic MPS I: 5-year follow-up. Pediatrics.

[42] McGill JJ, Inwood AC, Coman DJ, Lipke ML, de Lore D, Swiedler SJ (2010). Enzyme replacement therapy for mucopolysaccharidosis VI from 8 weeks of age--a sibling control study. Clin Genet.

[43] Furujo M, Kosuga M, Okuyama T (2017). Enzyme replacement therapy attenuates disease progression in two Japanese siblings with mucopolysaccharidosis type VI: 10-year follow up. Mol Genet Metab Rep.

[44] Franco JF, Soares DC, Torres LC, Leal GN, Cunha MT, Honjo RS, et al. Short Communication Impact of early enzyme-replacement therapy for mucopolysaccharidosis VI: results of a long-term follow-up of Brazilian siblings. Genet Mol Res. 2016;15(1). 10.4238/gmr.15017850. 10.4238/gmr.15017850 26910003

[45] Al-Sannaa NA, Bay L, Barbouth DS, Benhayoun Y, Goizet C, Guelbert N (2015). Early treatment with laronidase improves clinical outcomes in patients with attenuated MPS I: a retrospective case series analysis of nine sibships. Orphanet J Rare Dis.

[46] Lin SP, Lin HY, Wang TJ, Chang CY, Lin CH, Huang SF (2013). A pilot newborn screening program for Mucopolysaccharidosis type I in Taiwan. Orphanet J Rare Dis.

[47] Chuang CK, Lin HY, Wang TJ, Huang YH, Chan MJ, Liao HC (2018). Status of newborn screening and follow up investigations for Mucopolysaccharidoses I and II in Taiwan. Orphanet J Rare Dis.

[48] Colón C, Alvarez JV, Castaño C, Gutierrez-Solana LG, Marquez AM, O'Callaghan M (2017). A selective screening program for the early detection of mucopolysaccharidosis: results of the FIND project - a 2-year follow-up study. Medicine (Baltimore).

