# Supplementary material for: Cardiac features and effects of enzyme replacement therapy in Taiwanese patients with Mucopolysaccharidosis IVA
Source: Orphanet J Rare Dis. 2018 Aug 29;13:148. doi: 10.1186/s13023-018-0883-6 (PMC6114849; doi:10.1186/s13023-018-0883-6)
Supplement: Supplementary file 1 — Table S1. Baseline and follow-up echocardiographic assessments for valvular heart disease of seven Taiwanese patients with MPS IVA who received ERT for 3–6 years (severity score: 3: severe, 2: moderate, 1: mild, 0: normal). (DOCX 24 kb) [file 13023_2018_883_MOESM1_ESM.docx]

**Table S1** Baseline and follow-up echocardiographic assessments for valvular heart disease of seven Taiwanese patients with MPS IVA who received ERT for 3-6 years (severity score: 3: severe, 2: moderate, 1: mild, 0: normal).
